# Microbial fingerprinting detects intestinal microbiota dysbiosis in Zebrafish models with chemically-induced enterocolitis

**DOI:** 10.1186/1471-2180-13-289

**Published:** 2013-12-11

**Authors:** Qi He, Lin Wang, Fan Wang, Chenyang Wang, Chun Tang, Qiurong Li, Jieshou Li, Qingshun Zhao

**Affiliations:** 1 Research Institute of General Surgery, Jinling Hospital, School of Medicine, Nanjing University, No.305 East Zhongshan Road, Nanjing 210002, China; 2 Model Animal Research Center, MOE Key Laboratory of Model Animal for Disease Study, Nanjing University, Nanjing, China

**Keywords:** Zebrafish, Denaturing gradient gel electrophoresis, Trinitrobenzenesulfonic acid, Microbiota

## Abstract

**Background:**

Inflammatory bowel disease (IBD) involves a breakdown in interactions between the host immune response and the resident commensal microbiota. Recent studies have suggested gut physiology and pathology relevant to human IBD can be rapidly modeled in zebrafish larvae. The aim of this study was to investigate the dysbiosis of intestinal microbiota in zebrafish models with IBD-like enterocolitis using culture-independent techniques.

**Results:**

IBD-like enterocolitis was induced by exposing larval zebrafish to trinitrobenzenesulfonic acid (TNBS). Pathology was assessed by histology and immunofluorescence. Changes in intestinal microbiota were evaluated by denaturing gradient gel electrophoresis (DGGE) and the predominant bacterial composition was determined with DNA sequencing and BLAST and confirmed by real-time polymerase chain reaction. Larval zebrafish exposed to TNBS displayed intestinal-fold architecture disruption and inflammation reminiscent of human IBD. In this study, we defined a reduced biodiversity of gut bacterial community in TNBS-induced coliitis. The intestinal microbiota dysbiosis in zebrafish larvae with IBD-like colitis was characterized by an increased proportion of *Proteobacteria* (especially *Burkholderia*) and a decreased of Firmicutes(*Lactobacillus group*), which were significantly correlated with enterocolitis severity(Pearson correlation *p* < 0.01).

**Conclusions:**

This is the first description of intestinal microbiota dysbiosis in zebrafish IBD-like models, and these changes correlate with TNBS-induced enterocolitis. Prevention or reversal of this dysbiosis may be a viable option for reducing the incidence and severity of human IBD.

## Background

Inflammatory bowel disease (IBD), broadly classified into ulcerative colitis (UC) and Crohn’s disease (CD), is a chronic gastrointestinal (GI) illness of uncertain etiology with high morbidity and relapse. Symptoms range from abdominal pain, weight loss and diarrhea to ulceration, perforation and complete obstruction of the GI tract. Although the precise etiology of IBD remains unclear, several factors are believed to play a role in its development and progression, including host genotype, immune disequilibrium, the composition of microbial communities resident in the GI tract and environmental factors [1,2]. In particular, the interactions between intestinal epithelial damage and microbial incursion have become new research hotspots.

The human intestinal tract plays host to approximately 100 trillion microorganisms, with at least 15,000-36,000 bacterial species. The intestinal microbiota is now considered to be a functional organ associated with normal physiological processes, such as metabolism, immunological response and intestinal epithelium morphogenesis [3-5]. Thus, there are many areas of host health that can be compromised when the microbiota is drastically altered. IBD clearly involves a breakdown in interactions between the host immune response and the resident commensal microbiota. Several investigators have documented changes in the gut microbiota associated with IBD, especially a dramatically reduced diversity in the phylum *Firmicutes* and concomitant increase in *Proteobacteria*[6-8]. In humans, a therapeutic strategy called fecal bacteriotherapy involving transfer of fecal material from a healthy donor to an IBD patient has successfully ameliorated the disease [9,10]. That the restoration of microbial diversity is effective suggests the intestinal microbiota alteration may play a key role in disease pathogenesis. However, our knowledge of the microbiota shifts associated with IBD is far from complete, and it remains a question whether these changes are responsible for the origin of IBD, or alternatively, a direct or indirect consequence.

Murine models, for example, IL-10 deficient (IL-10−/−) mice and dextran sodium sulfate (DSS)-treated mice, have contributed enormously to understand the pathogenesis of IBD. Previous reports on DSS-induced colitis in murine models revealed that oral DSS-induced mucosal injury is more extensive in animals with commensal bacterial depletion compared to conventionalize counterparts. Contradictory data was seen in IL-10 deficient (IL-10−/−) mice, showing that IL-10−/− mice fail to develop spontaneous colitis if reared in germ-free conditions [11-13]. The difference may be caused by a variety of pathogenic mechanism, however, the research is limited by the time, cost and ethics and a new animal model is in badly need. The zebrafish model as an established developmental biology model has recently come to the fore in the study of developmental biology and disease processes. Fleming et al. developed an IBD-like model in zebrafish larvae using 2, 4, 6-trinitrobenzenesulfonic acid (TNBS), which enable study of host-bacterial interactions in detail in IBD processes [14,15]. The zebrafish digestive tract is similar to that of mammals in its development, organization and function, and observation of the larvae gut following induction of IBD reveals region specific disease changes with biological, pathological and clinical relevance to the human condition [14-17]. Additionally, the zebrafish environment is relatively easy to manipulate and embryos can conveniently be produced in large numbers. Finally, the intestines of the zebrafish can be embedded in whole for analysis.

Zebrafish are well suited for studying host-bacterial interactions as they have innate and adaptive immune systems similar to higher vertebrates [18]. Comparative metagenomic profiling of zebrafish and mouse gut microbiota revealed that they share six bacterial divisions, including *Proteobacteria*, *Firmicutes*, *Bacteroidetes*, *Verrucomicrobia*, *Actinobacteria* and *Planctomycetes* divisions [19]. Besides, microarray analysis of gnotobiotic zebrafish has revealed transcriptional alterations in response to the microbiota that consistent with mammals, demonstrating an evolutionarily conserved role of the gut microbiota in vertebrate development [20,21]. Moreover, the resident commensal microbiota in both fish and mice provide similar functions in the gut: they ferment polysaccharides to short-chain fatty acids (SCFAs) and play an important role in defense against pathogenic infection [21,22]. In addition, studies in zebrafish gut differentiation show that in the absence of microbiota, the larvae gut is arrested in specific aspects of differentiation and altered in specific aspects of its function, which can be reversed by the introduction of bacteria later in development [5]. Another study revealed alterations on gut microbiota after feeding the zebrafish dietary probiotic *Lactobacillus rhamnosus* for 10 days, which has significant effects on the reproductive physiology [23]. All of this suggests that the microbiota in zebrafish gut may play the same role in disease pathogenesis as in mammals.

The aim of the work reported here was to carry out a molecular analysis on the composition of the intestinal microbiota in zebrafish larvae with TNBS-induced IBD-like colitis applying PCR-denaturing gradient gel electrophoresis (DGGE). A range of TNBS doses and exposure times were investigated in order to find out whether the intestinal epithelial damage and microbiota alternations in colitis processes are dose and time dependent fashion. Furthermore, we aimed to identify specific bacterial species of the gut microbiota that could be associated with the pathogenesis of colitis in zebrafish by DNA sequence analysis. Consequently, we also revealed the establishment of the resident microbiota in larval zebrafish gut from individuals of developing fish from 4 dpf to 8 dpf. Within the present work, we analyzed the zebrafish TNBS-induced enterocolitis in greater detail and first defined the changes of the intestinal microbiota in zebrafish IBD-like models, which might provide novel knowledge on the role of intestinal bacterial dysbiosis in IBD pathogenesis and show technical feasibility of studying host-bacterial interactions in IBD processes.

## Results

### Pathological changes in TNBS-induced enterocolitis

**Figure 1:**
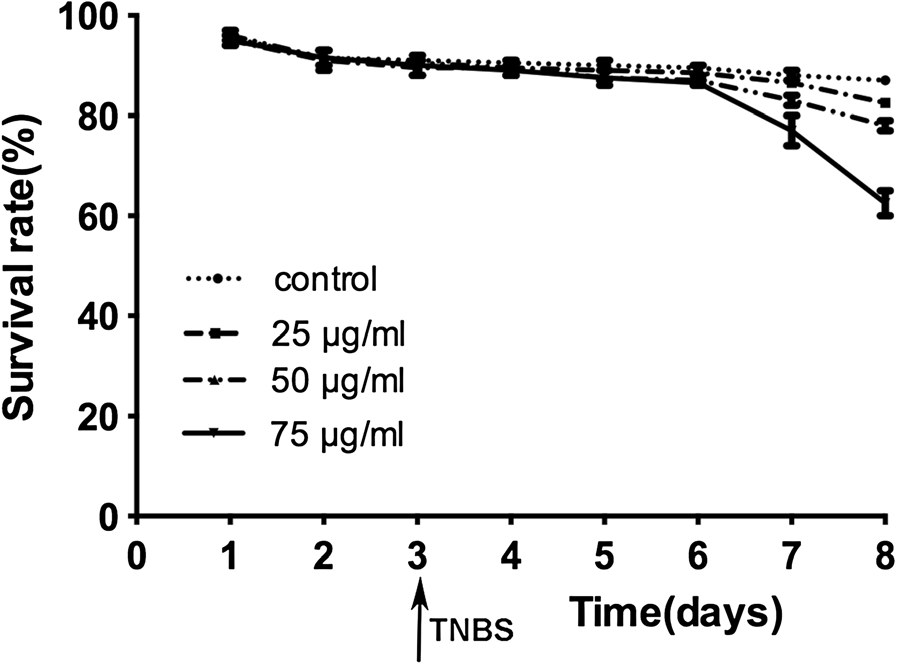
**Effect of different 2, 4, 6-trinitrobenzenesulfonic acid (TNBS) concentrations (0, 25, 50 and 75 μg/ml) in the cumulative survival rate.** Zebrafish were exposed to TNBS from 3 days post fertilization (dpf). Results are representative of three independent experiments. Values are presented as mean ± SEM.

The record of the dose-dependent and time-course survivorship of the embryos/larvae is shown in Figure 1. The treatment of TNBS started from 3 days post fertilization (dpf) until harvest at 4, 6 or 8 dpf in each TNBS-exposed group. Before 8 dpf, there was no significant difference in the percentage of survivorship in any of the TNBS-exposed groups compared to the controls. At TNBS concentrations of 25 and 50 μg/ml, no significant increase in mortality was observed over the whole exposure time, whereas a slight increase (*p*<0.05) in mortality was observed in the dose of 75 μg/ml TNBS.

**Figure 2:**
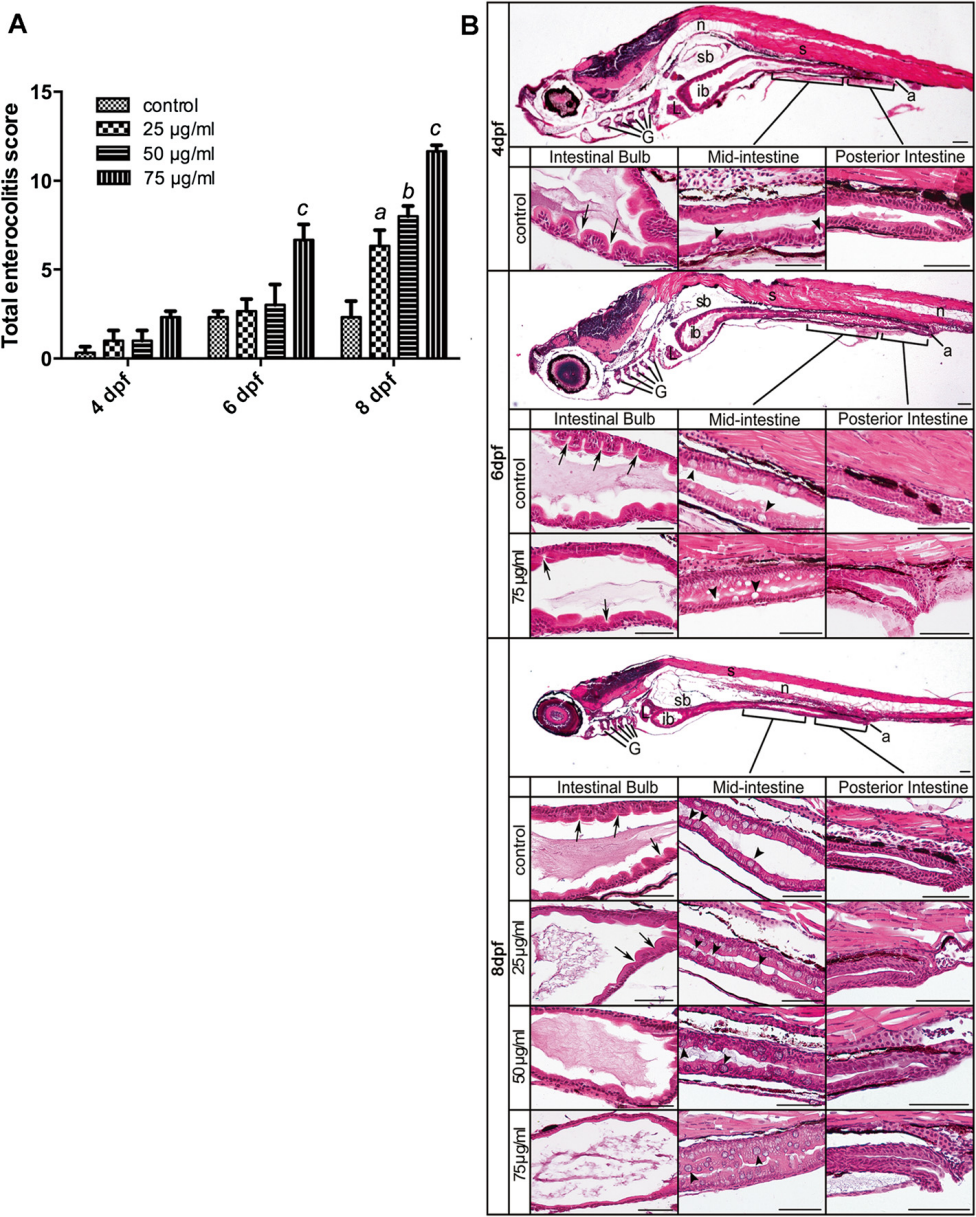
**Histological analysis of TNBS-induced enterocolitis. A**: Total enterocolitis score of larval zebrafish exposed to different TNBS concentrations (0, 25, 50 and 75 μg/ml) at 4, 6 and 8 dpf. The scores were quantified by a blinded scorer. For each score, a total of 30 folds (10 per intestinal segment) were evaluated per intestine and 6 intestines were evaluated for each experimental group from three independent experiments. All error bars represent as mean ± SEM. n=6 larvae per group, ^*a*^Indicates a significant difference (*p*<0.05) between TNBS-exposed group (25 μg/ml) and the control, ^*b*^Indicates a significant difference (*p*<0.05) between TNBS-exposed group (50 μg/ml) and the control, ^*c*^Indicates a significant difference (*p*<0.05) between TNBS-exposed group (75 μg/ml) and the control, ^*d*^Indicates a significant difference (*p*<0.05) between control groups at 6 dpf and 4 dpf, ^*e*^Indicates a significant difference (*p*<0.05) between control groups at 8 dpf and 4 dpf. **B**: Representative haematoxylin-eosin stained sagittal sections of the whole intestine tact and regions of the intestinal bulb, the mid-intestine and the posterior intestine from the statistically significant groups taken at 4, 6 and 8 dpf. In the segment of the intestinal bulb (ib), the lumen expands and the depth of epithelial folds is progressively reduced during TNBS exposure (arrows). The mid-intestine is demarcated by the presence of goblet cells and shows increased numbers with TNBS treatment (arrowheads). No significant changes are shown in the posterior intestine region between control and TNBS-exposed samples. a, anus; ib, intestinal bulb; G, gill arches; L, liver; sb, swim bladder; n, notochord; s, somite. Scale bars, 50 μm.

For evaluation of enterocolitis changes caused by TNBS exposure, a simple scoring system was devised (Table 1). Intestinal bulb, mid-intestine, and posterior intestine were assessed separately. Total enterocolitis score representing the cumulative values of these separate parameters for all 3 segments of the intestine is shown in Figure 2A. Zebrafish collected at 4 dpf showed no significant difference between TNBS-treated and control samples. However, changes were first observed at 6 dpf in the high dose of 75 μg/ml TNBS exposed larvae (7, compared with 0 in the control group). At 8 dpf, there was a significant dose-dependent increase in the enterocolitis score of TNBS-exposed groups (6, 8 and 12 in the dose of 25, 50 and 75 μg/ml, respectively), as compared with the score of 3 in the control. It demonstrated administration of TNBS to the embryo medium was able to induce enterocolitis.

**Table 1:** Enterocolitis score system for histology evaluation

**Numerical score**	**Intestinal-fold architecture disruption**			**Goblet cell appearance**
	**Impaired epithelial integrity**	**Expanded clefts/reduced projections**	**Expanded gut lumen**	
0	Normal	Normal	Normal	Normal
1	Slight disruption	Slight disruption	Slight expansion	Increased in number
2	Moderate disruption	Moderate disruption	Moderate expansion	Increased in number and different in sizes
3	Severe disruption	Severe disruption	Severe expansion	Severe morphological changes

Representative pictures of the statistically significant groups are shown in Figure 2B. In the intestine bulb, the epithelium of control samples was characterized by projections and clefts, whereas in TNBS-treated samples the epithelium appeared smooth and the lumen was expanded. In the mid-intestine region, higher numbers of goblet cells were observed in TNBS-exposed fish compared with controls. Histological analysis did not show epithelial architecture disruption in the posterior intestine of both control and TNBS-exposed groups. In addition, goblet cells were observed in the regions of intestinal bulb and posterior intestine of larvae exposed to TNBS, while the presence of goblet cells remained restricted to the mid-intestine in the control.

**Figure 3:**
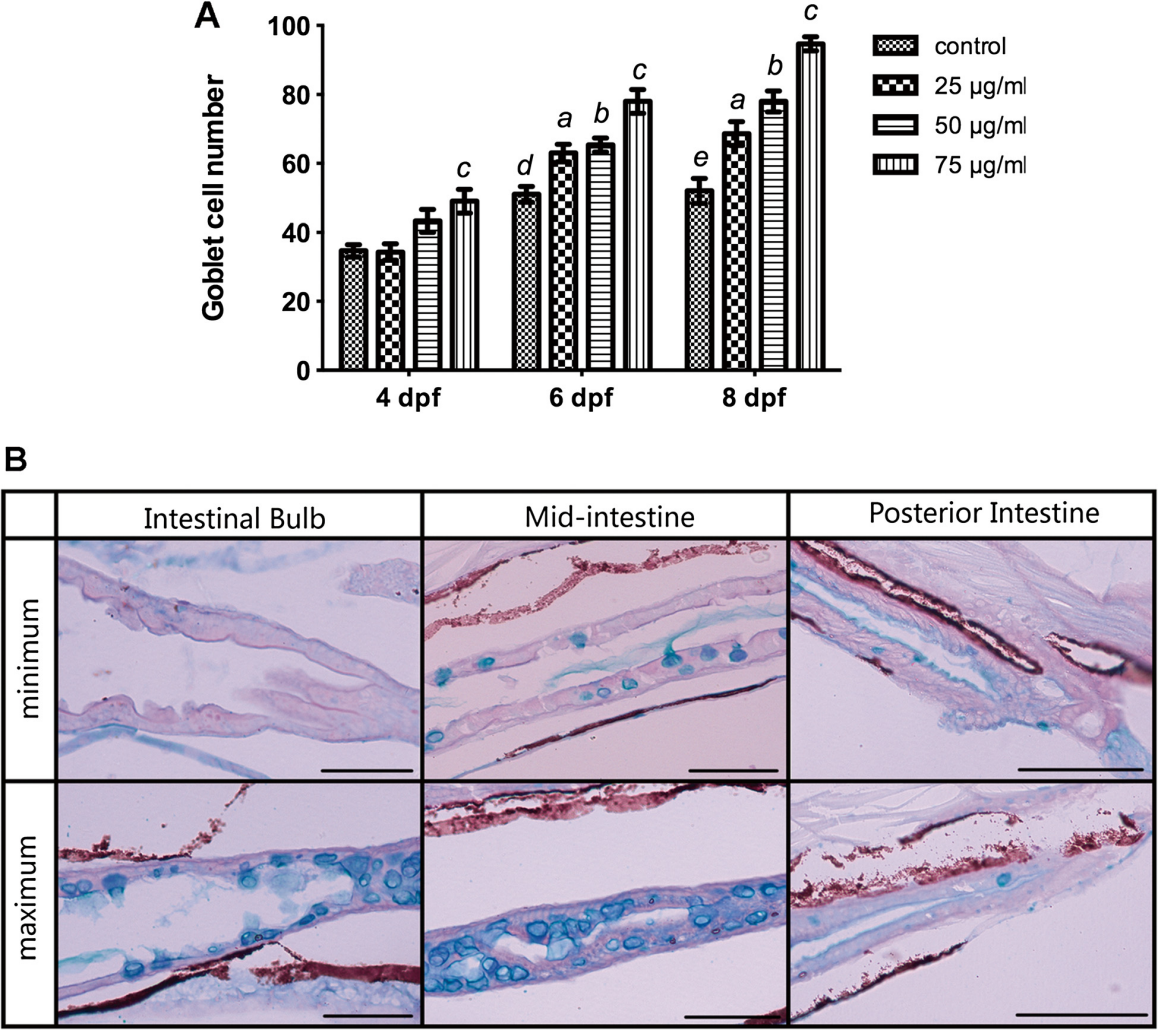
**Quantification of goblet cells stained with Alcian blue and periodic acid Schiff reagent (AB–PAS). A**: Goblet cell number increased with increasing concentrations of TNBS over time. All error bars represent as mean ± SEM. n=10 larvae per group, ^*a*^Indicates a significant difference (*p*<0.05) between TNBS-exposed group (25 μg/ml) and the control, ^*b*^Indicates a significant difference (*p*<0.05) between TNBS-exposed group (50 μg/ml) and the control, ^*c*^Indicates a significant difference (*p*<0.05) between TNBS-exposed group (75 μg/ml) and the control, ^*d*^Indicates a significant difference (*p*<0.05) between control groups at 6 dpf and 4 dpf, ^*e*^Indicates a significant difference (*p*<0.05) between control groups at 8 dpf and 4 dpf. **B**: Representative pictures of maximum and minimum numbers of goblet cells in the intestinal bulb, the mid-intestine and the posterior intestine. Histochemical staining with AB–PAS demonstrates that goblet cells continue to synthesize acidic mucins.

The increase in goblet cells observed in TNBS-exposed larvae was further detected using AB-PAS staining as described above. As it is shown in Figure 3A, the number of goblet cells significantly increased with time and in a dose-dependent pattern. Representative pictures of maximum and minimum numbers of goblet cells in all 3 regions of the intestinal tract were shown in Figure 3B.

### Inflammatory cytokine production in larvae exposed to TNBS

**Figure 4:**
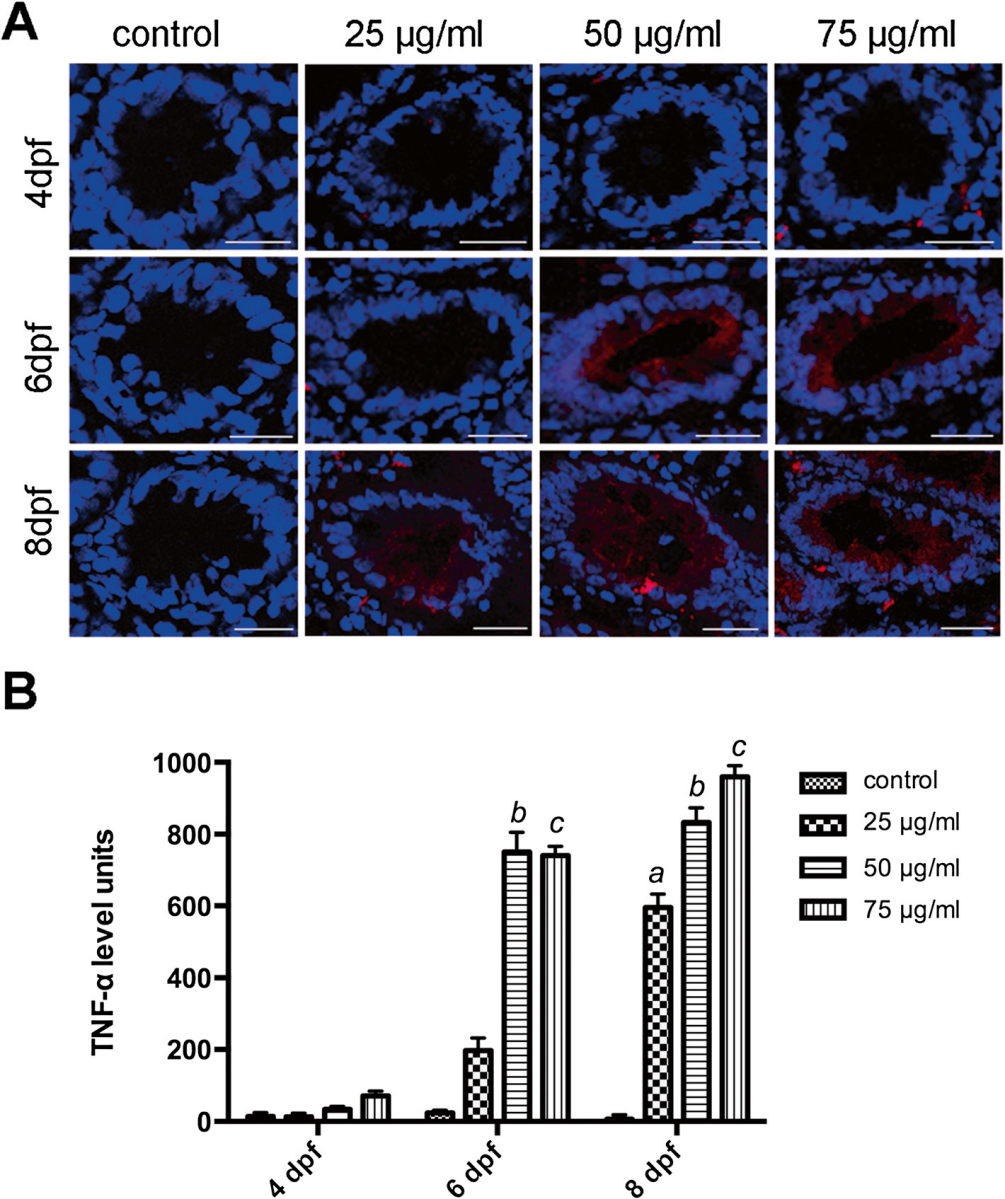
**Immunofluorescence analysis of TNF-α expression in gut. A**: TNF-α expression was stimulated in larvae exposed to TNBS. TNF-α staining (red) and DAPI staining (blue) images were visualized by confocal laser scanning microscopy. Bars: 25 μm. **B**: TNF-α immunofluorescence levels increased with increasing concentrations of TNBS over time. All error bars represent as mean ± SEM, n=13–16 sections per group, ^*a*^Indicates a significant difference (*p*<0.05) between TNBS-exposed group (25 μg/ml) and the control, ^*b*^Indicates a significant difference (*p*<0.05) between TNBS-exposed group (50 μg/ml) and the control, ^*c*^Indicates a significant difference (*p*<0.05) between TNBS-exposed group (75 μg/ml) and the control, ^*d*^Indicates a significant difference (*p*<0.05) between control groups at 6 dpf and 4 dpf, ^*e*^Indicates a significant difference (*p*<0.05) between control groups at 8 dpf and 4 dpf.

**Figure 5:**
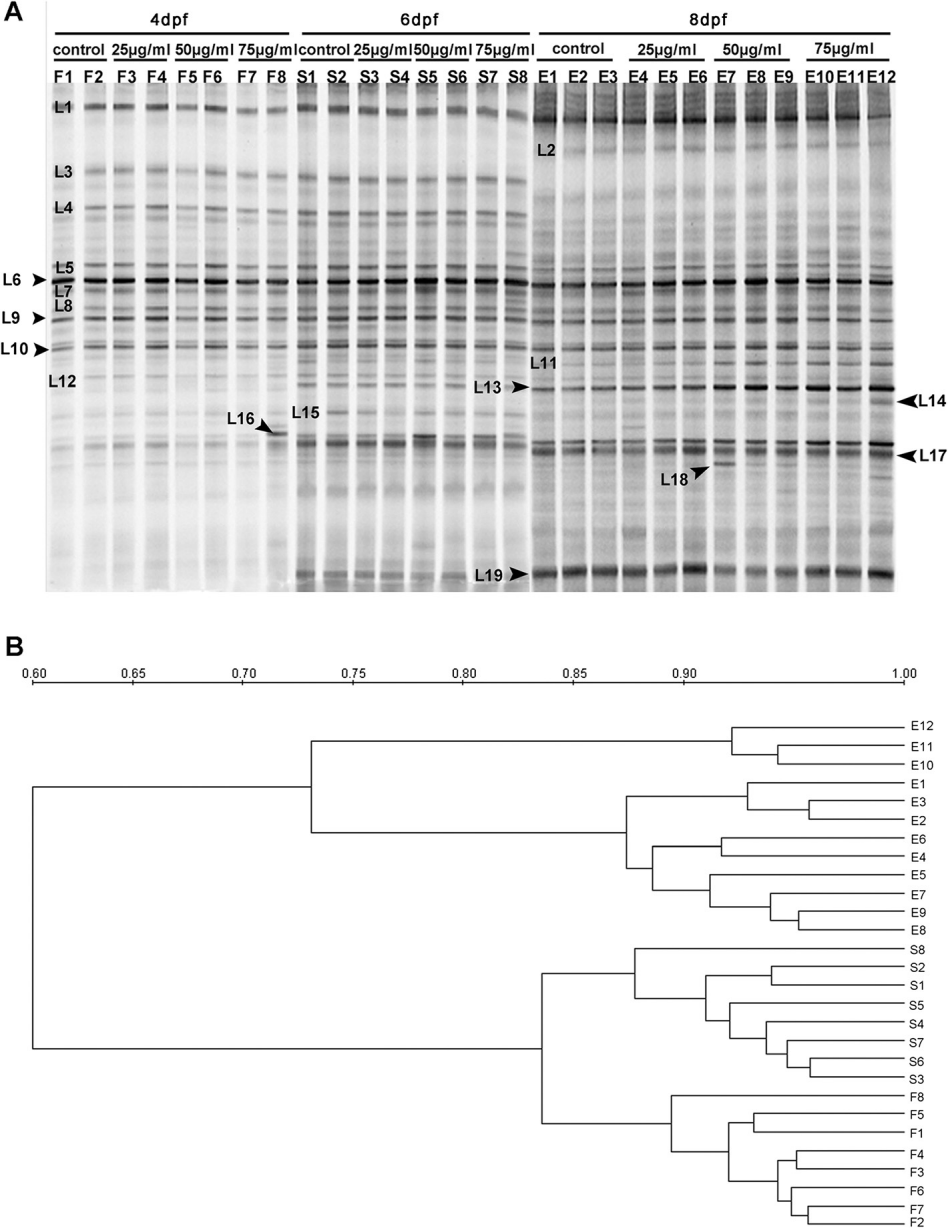
**Intestinal microbiota dysbiosis in zebrafish with TNBS-induced enterocolitis. A**: Representative denaturing gradient gel electrophoresis (DGGE) profiles generated for the gut microbiota community of zebrafish with TNBS-exposure and without it (control) collected at 4, 6 and 8 dpf. **B**: Dendrogram constructed with intestinal microbiota community fingerprints based on cluster analysis by unweighted pair group method using arithmetic averages (UPGMA).

TNF-α expression was examined using immunofluorescence to measure inflammatory reactions in larval zebrafish exposed to TNBS. In our study, TNF-α appeared as red fluorescent light in plasma around the nucleus within the intestinal epithelium (Figure 4A). In the control groups, TNF-α staining is absent from the gut (Figure 4A and B). However, TNF-α expression was stimulated significantly with increasing concentrations of TNBS (Figure 4B). In addition, larvae exposed to the same dose of TNBS, TNF-α immunofluorescence levels increased as the exposure time grew (Figure 5B). It proved TNBS exposure primarily evoked an inflammatory response within the intestine dose and time dependently.

### Shifts in intestinal microbiota during TNBS-induced inflammation

The PCR-DGGE fingerprints showed changes of the composition and diversity in gut microbiota of the twelve groups of fish (Figure 5A). The first eight lanes represent the DGGE profiles of control and TNBS-exposed fish harvested at 4 dpf, whereas the lanes 9 to 16 represent the profiles of fish at 6 dpf and the last twelve lanes are the profiles at 8 dpf. At each of the time point, the gel shows the DGGE profiles of 4 groups: control (F1-F2, S1-S2, E1-E3), 25 μg/ml TNBS-exposed (F3-F4, S3-S4, E4-E6), 50 μg/ml TNBS-exposed (F5-F6, S5-S6, E7-E9) and 75 μg/ml TNBS-exposed (F7-F8, S7-S8, E10-E12).

The dendrogram based on DGGE banding similarity patterns showed that samples from different time points were separated into three different clusters (Figure 5B), indicating the establishment of the gut microbiota during zebrafish development from 4 to 8 dpf. At 8 pdf, the microbial composition in the control and TNBS-exposed groups especially the 75 μg/ml TNBS-exposed group had a significant variation, whereas at 4 and 6 dpf, the community profiles were not clearly distinct. It revealed TNBS exposure resulted in intestinal microbiota alteration by 8 pdf.

**Figure 6:**
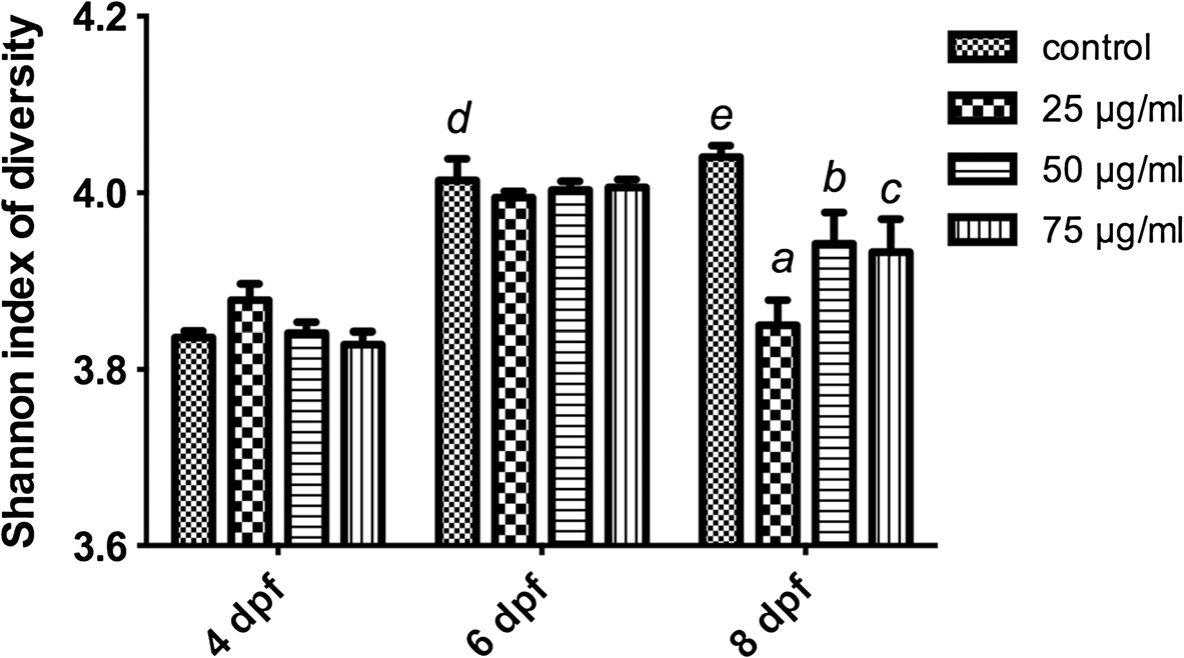
**Biodiversity of microbiota composition in zebrafish with TNBS-induced IBD.** All error bars represent as mean ± SEM. n=6 samples per group, ^*a*^Indicates a significant difference (*p*<0.05) between TNBS-exposed group (25 μg/ml) and the control, ^*b*^Indicates a significant difference (*p*<0.05) between TNBS-exposed group (50 μg/ml) and the control, ^*c*^Indicates a significant difference (*p*<0.05) between TNBS-exposed group (75 μg/ml) and the control, ^*d*^Indicates a significant difference (*p*<0.05) between control groups at 6 dpf and 4 dpf, ^*e*^Indicates a significant difference (*p*<0.05) between control groups at 8 dpf and 4 dpf.

The alternations of Shannon-Wiener diversity indices according to the intensity of bands were showed in Figure 6. As we can see, during the bacterial colonization of the zebrafish gut from 4 to 8 dpf, the biodiversity of intestinal microbiota was increased. Meanwhile, larvae exposed to TNBS had a lower community diversity of gut bacteria compared to control group at 8 dpf.

### Bacterial species associated with inflammatory disorder

**Figure 7:**
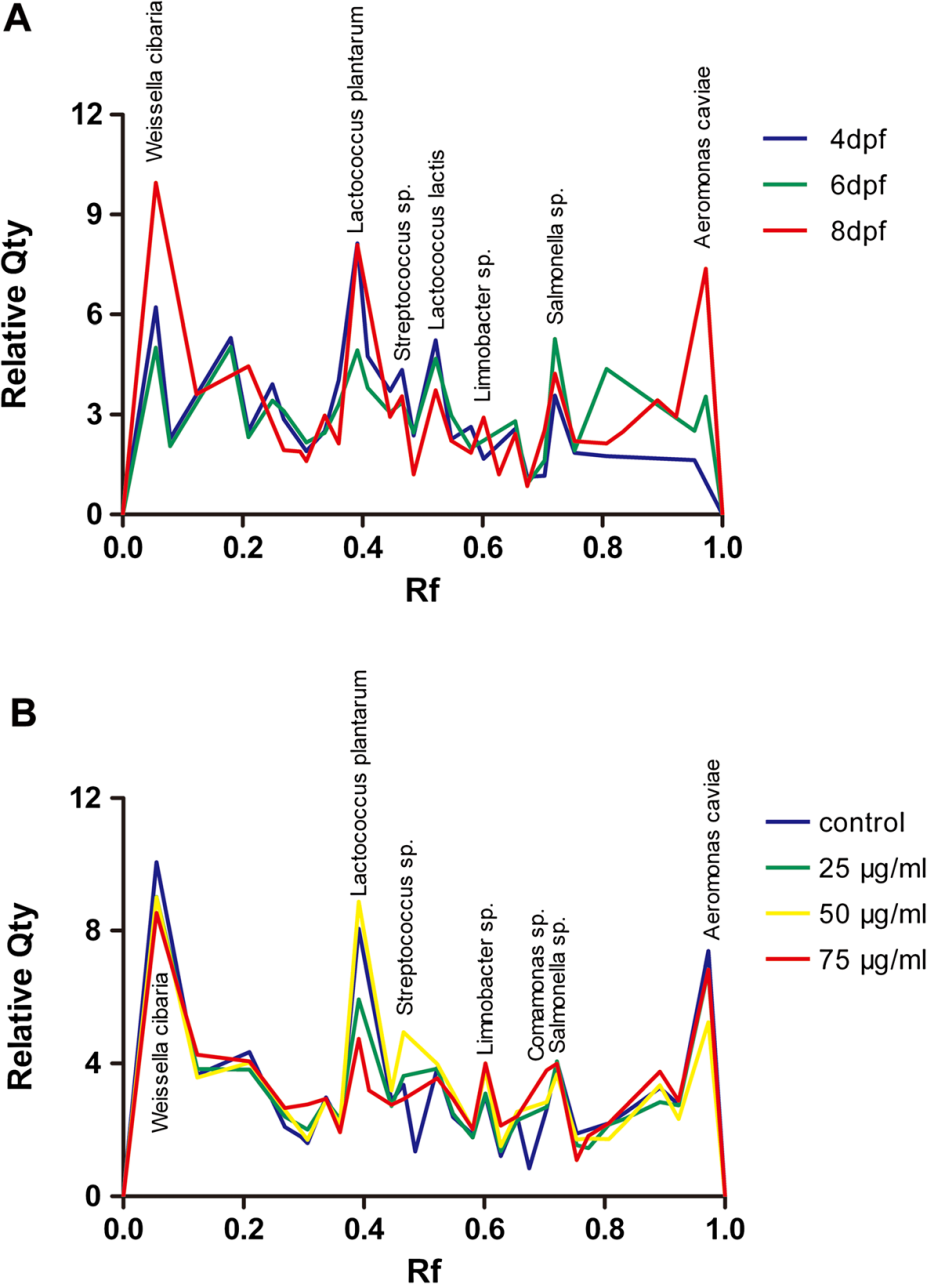
**The relative abundance of predominant bacteria in zebrafish intestine. A**: The mean richness of DGGE bands from the control samples collected at 4, 6 and 8 dpf. **B**: The mean richness of DGGE bands from the samples exposed to different TNBS concentrations (0, 25, 50 and 75 μg/ml) collected at 8 dpf. The staining intensity of fragments was expressed as a proportion (%) of the sum of all fragments in the same lane. Rf, relative front.

In order to define the key members of intestinal microbiota that likely contributed to the pathogenesis of TNBS-induced inflammatory disorder, we further identified the alteration of the dominant bacterial species in zebrafish gastrointestinal tract. Nineteen sequences of 16S rRNA gene fragments were obtained and sequenced. These genes were assigned to 19 bacterial phylotypes based on the highest sequence similarity (95–100%) matched to GenBank sequences obtained by BLAST analysis (Figure 5A, Table 2). We next quantified the relative abundance of fragments in DGGE profiles of the 19 bacterial phylotypes (Figure 7).

**Table 2:** The sequence analysis of DGGE bands from the zebrafish GI tract

**Phylum**	**Class**	**Family**	**Closest relative**	**Strain**	**GenBank accession no.**	**% similarity**	**Isolates (Band)**
Firmicutes		Leuconostocaceae	Weissella cibaria	AC26	KF515539	100	L1
			Leuconostoc holzapfelii	IMAU62126	KF515541	97	L3
			Lactococcus raffinolactis	S56-2	KF515542	100	L4
			Lactococcus lactis	LD11	KF515543	100	L5
			Lactococcus plantarum	DSM 20686	KF515544	99	L6
			Lactococcus lactis	SS11A	KF515548	99	L10
		Veillonellaceae	Veillonella sp.	S101	KF515546	100	L8
		Streptococcaceae	Streptococcus sp.	LVRI-122	KF515547	100	L9
Proteobacteria	β-Proteobacteria	Burkholderiaceae	Limnobacter sp.	F3	KF515551	98	L13
		Comamonadaceae	Comamonas sp.	SB20	KF515554	99	L16
	γ-proteobacteria	Sinobacteraceae	Hydrocarboniphaga daqingensis	B2-9	KF515549	97	L11
		Moraxellaceae	Acinetobacter sp.	CHE4-1	KF515550	100	L12
		Sphingomonadaceae	Citrobacter freundii	T7	KF515552	95	L14
		Enterobacteriaceae	Pantoea rodasii	ORC6	KF515553	100	L15
			Salmonella sp.	Co9936	KF515555	96	L17
			Citrobacter werkmanii	HTGC	KF515556	98	L18
		Aeromonadaceae	Aeromonas caviae	BAB556	KF515557	96	L19
			Uncultured bacterium	S2-2-660	KF515540	100	L2
			Uncultured bacterium	B2-2	KF515545	100	L7

As shown in Figure 7A, the composition of the bacterial community in larvae digestive tract changed over time to become dominated by the bacterial phyla of *Proteobacteria* and *Firmicutes*. In particular, the proportions of *Proteobacteria* phylum, including *Hydrocarboniphaga daqingensis* (L11), *Limnobacter sp.* (L13), *Comamonas sp.* (L16), *Salmonella sp.* (L17) and *Aeromonas caviae* (L19), were dramatically increased from 4 dpf to 8 dpf (*p*<0.01).

Meanwhile, the significant alterations in the abundance of the 19 bacterial phylotypes between the TNBS-exposed groups and controls at 8 dpf were revealed (Figure 7B). The sections of *Proteobacteria* , such as *Hydrocarboniphaga daqingensis*(L11), *Limnobacter sp.* (L13), *Citrobacter freundii* (L14), *Comamonas sp.* (L16) and *Salmonella sp.* (L17), showed an increase in relative richness in the gut microbiota of zebrafish exposed to TNBS as comparison with the control group (*p*<0.01). However, *Citrobacter werkmanii* (L18) was less abundant in TNBS-exposed groups than in the control (*p*<0.05). In addition, *Firmicutes* bacteria consisting of *Lactococcus plantarum* (L6), and *Streptococcus sp.* (L9) were less present in TNBS-exposed fish (*p*<0.05).

**Figure 8:**
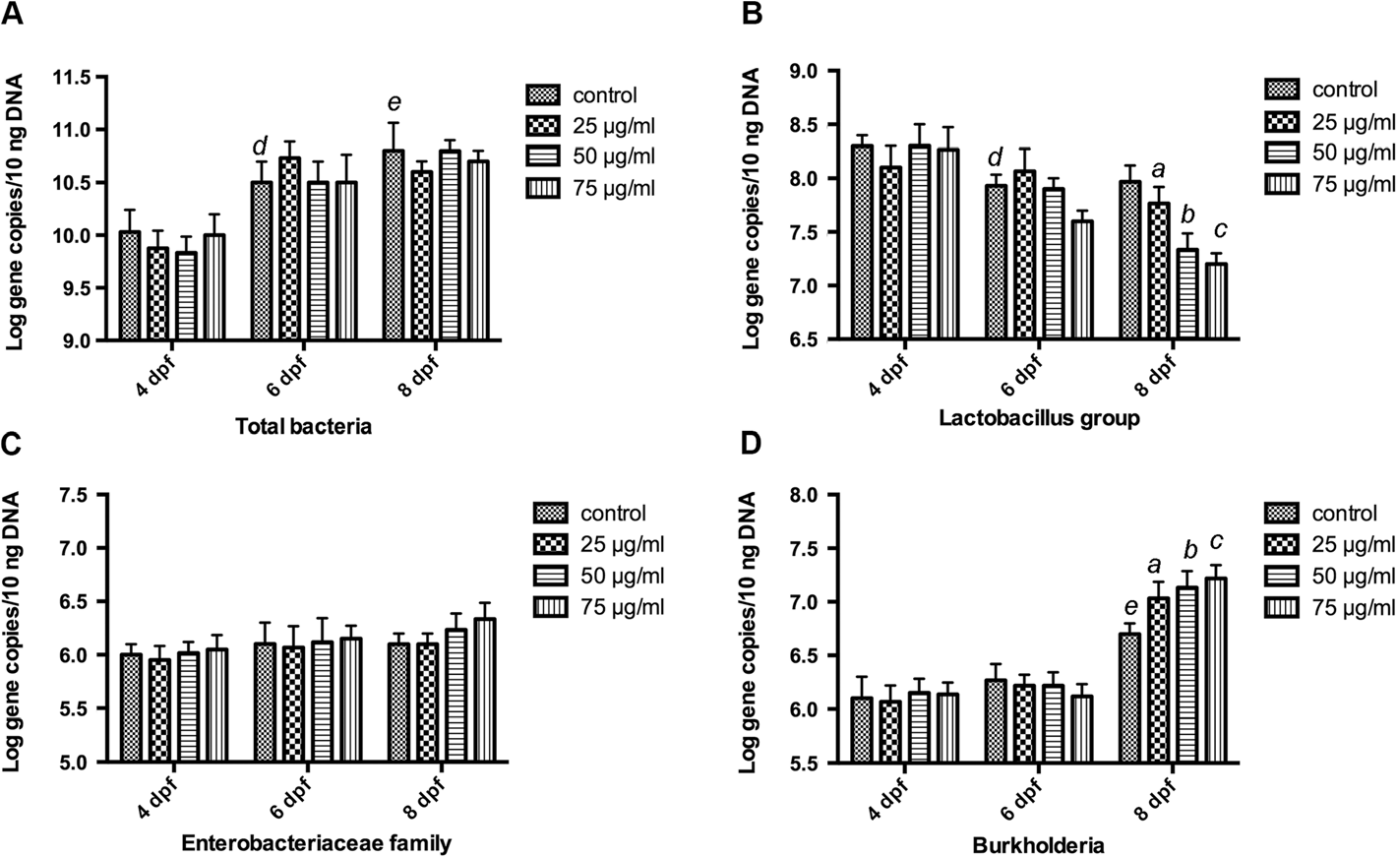
**Quantitative analysis of characteristic bacterial species.** The relative quantity of specific groups of bacteria was determined by real-time PCR of 16S rRNA gene of **(A)***Total bacteria*, **(B)***Lactobacillus group*, **(C)***Burkholderia* and **(D)***Enterobacteriaceae family*. All reactions were performed in triplicate. Specific bacteria 16S rRNA gene amount was normalized to total bacteria 16S rRNA. Quantification values were represented as mean ± SEM log 16S rRNA gene copies per 10 ng of bacterial genomic DNA. ^*a*^Indicates a significant difference (*p*<0.05) between TNBS-exposed group (25 μg/ml) and the control, ^*b*^Indicates a significant difference (*p*<0.05) between TNBS-exposed group (50 μg/ml) and the control, ^*c*^Indicates a significant difference (*p*<0.05) between TNBS-exposed group (75 μg/ml) and the control, ^*d*^Indicates a significant difference (*p*<0.05) between control groups at 6 dpf and 4 dpf, ^*e*^Indicates a significant difference (*p*<0.05) between control groups at 8 dpf and 4 dpf.

Quantitative real-time PCR was performed to verify the changes found by DGGE. The toltal number of bacteria was significantly increased from 4 dpf to 8 dpf (*p*<0.001, Figure 8A). However, no differences in total number of bacteria were found between TNBS-treated groups and the control. At 8 dpf, *Lactobacillus group* was significantly reduced in the TNBS-exposed groups (Figure 8B). Numbers of *Burkholderia* increased significantly (Figure 8D), but not *Enterobacteriaceae family* (Figure 8C). Which was consistent with the DGGE result.

### Enterocolitis severity and TNF-α expression correlate with the composition in gut microbiota

**Figure 9:**
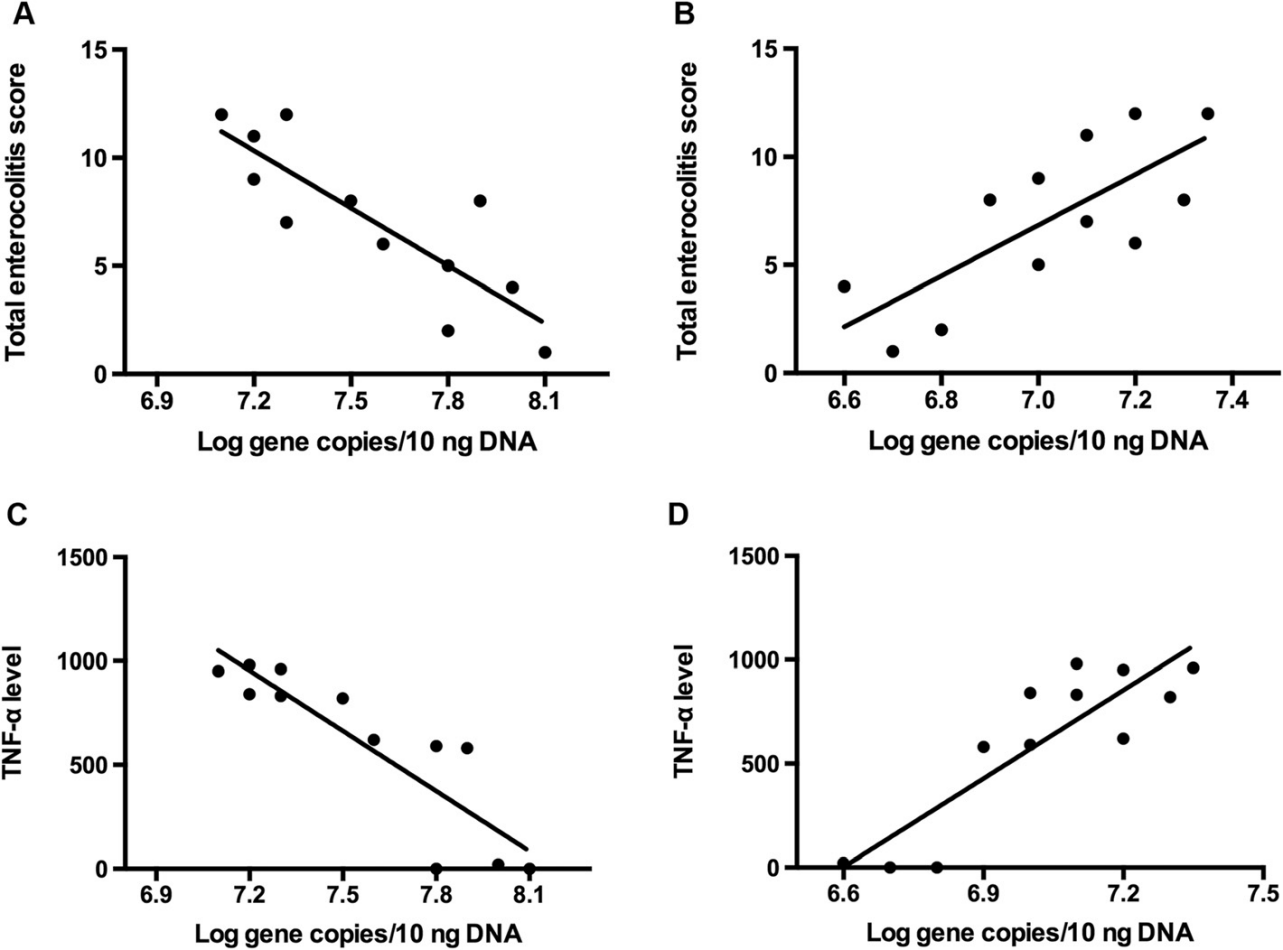
**Total enterocolitis score and TNF-α expression level correlate with the composition of intestinal microbiota. A**: Pearson correlation between total enterocolitis score and *Lactobacillus group* 16S rRNA gene copies, *p*=0.0045. **B**: Pearson correlation between total enterocolitis score and *Burkholderia* 16S rRNA gene copies, *p*=0.006. **C**: Pearson correlation between TNF-α expression levels and *Lactobacillus group* 16S rRNA gene copies, *p*=0.002. **D**: Pearson correlation between TNF-α expression levels and *Burkholderia* 16S rRNA gene copies, *p*=0.002.

We had observed the severity of enterocolitis in TNBS-treated zebrafish increased in a dose-dependent pattern at 8 dpf as compared with contols (Figure 2), whereas the abundance of *Proteobacteria* (especially *Burkholderia*) dramatically increased and the proportion of *Firmicutes* (*Lactobacillus group*) decreased significantly (Figure 8). We may predict that colitis severity would correlate with TNBS-induced changes in composition of gut bacteria. Accordingly, we calculated the correlation between enterocolitis scores and the density of *Burkholderia* and *Lactobacillus group* separately by Pearson correlation analysis. We found that the colitis scores correlated with the abundance of *Burkholderia* (*p*=0.0045, Figure 9A) and the richness of *Lactobacillus group* (*p*=0.006, Figure 9B). These findings demonstrate that TNBS-induced enterocolitis correlate with changes in the composition and density of the gut microbiota.

In the same way, we generated the correlation between TNF-α expression and TNBS-induced alterations in of gut microbiota. It came to the same conclusion that TNF-α expression correlated with the density of *Burkholderia* and *Lactobacillus group* and intestinal microbiota diversity, separately (Figure 9C, D).

### Phylogenetic analysis of the predominant bacteria

**Figure 10:**
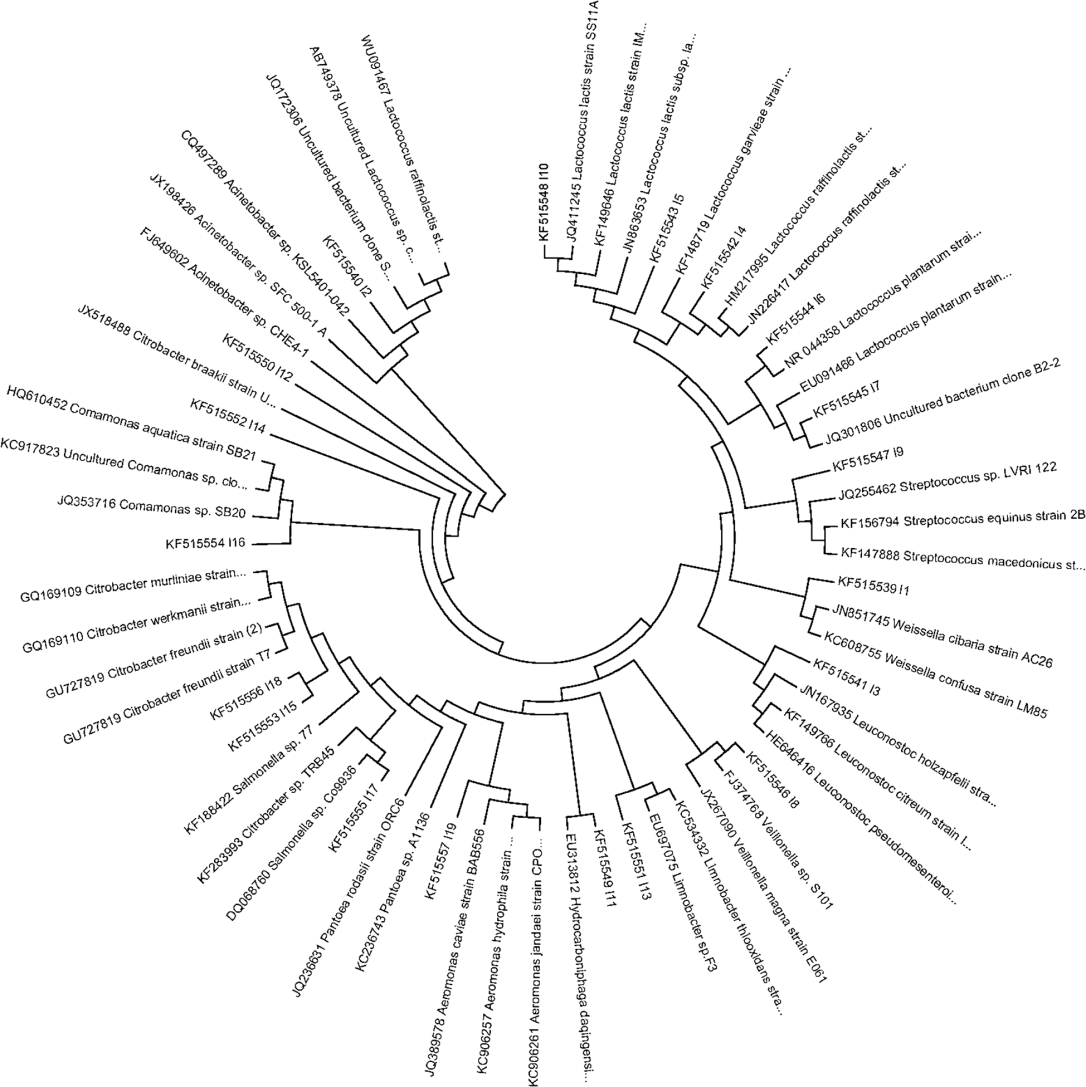
**Phylogenetic analysis based on partial 16S rRNA gene sequences of predominant bacterial species in the gut of zebrafish obtained from this study and some of those available in GenBank.** Identification and GenBank accession numbers are indicated for each sample. The evolutionary history was inferred using the Neighbor-Joining method. The optimal tree with the sum of branch length = 4.46466368 is shown. The evolutionary distances were computed using the Maximum Composite Likelihood method and are in the units of the number of base substitutions per site. Codon positions included were 1st+2nd+3rd+Noncoding. All positions containing gaps and missing data were eliminated from the dataset (Complete deletion option). There were a total of 62 positions in the final dataset. Phylogenetic analyses were conducted in MEGA4.

A phylogenetic tree depicting the evolutionary correlations among 19 bacteria and some of their relatives available in GenBank (similarity>95%), inferred on the basis of aligned 16S rDNA sequences, is shown in Figure 10. It showed that the dominant sequences from the zebrafish gut were phylogenetically clustered into 2 phylum: *Firmicutes* (total 9 sequences: 7 of *Lactobacillales*, 1 of *Clostridiales* and 1 of *Uncultured bacterium*) and *Proteobacteria *(total 10 sequences: 7 of *γ-Proteobacteria*, 2 of *β-Proteobacteria* and 1 of *Uncultured bacterium*).

## Discussion

In the present study, we established a zebrafish model organism to mimic human IBD using TNBS originally described by Fleming et al. It is confirmed that gut physiology and pathology relevant to this human disease state can be rapidly modeled following TNBS exposure, including intestinal epithelial damage, increase in goblet cells, production of inflammatory cytokines and intestinal microbiota dysbiosis. From the histological assessment of damage severity in the gut it was apparent that all larvae from the healthy control group showed no overt features of enterocolitis, while larvae exposed to TNBS exhibited pathological features consistent with enterocolitis time- and dose- dependently. The results present a detailed characterization of the development of intestinal inflammation in TNBS-treated larval zebrafish and establish a basis for using zebrafish to explore unique bacterial communities involved in the pathogenesis of IBD.

The aim of this study was to characterize the intestinal microbiota dysbiosis in the gut of zebrafish with IBD induced by TNBS, and to identify individual bacterial species that contribute to these dysbiosis. It is widely believed that IBD involves a breakdown in relations between the host immune response and microbial population resident in the GI tract. Reduced richness or diversity of bacterial species has been reported widely in patients with ulcerative colitis and Crohn’s disease, as well as in animal models with IBD, which was consistent with our observation [6,8]. It could be hypothesized that, from its gut microbial community composition, the healthy larvae may have been more likely to format a stable micro-ecosystem with the intestinal environment, the gut epithelium and the mucosal immune system, therefore, less susceptible of developing IBD. Most studies suggest that the gut microbiota is an important factor in the pathogenesis of IBD, however, little is known about the contributions of particular intestinal species to health and disease.

Recently, increasingly molecular profiling techniques are being employed for the detection and characterization of the unculturable bacteria in the human colon. Studies based on DGGE have shown a faecal microbiota dysbiosis signature associated with CD, characterised by a decreased presence of *Faecalibacterium prausnitzii*, *Bifidobacterium adolescentis*, *Dialister invisus*, an unknown *Clostridium sp.* and an increased presence of *Ruminococcus gnavus*[24]. Others revealed that *Bacteroides vulgatus*, *Bacteroides uniformis*, and *Parabacteroides sp.* were more commonly present at higher levels in healthy controls than in UC or IBD patients [25]. The changes of the intestinal microbiota in IBD patients were not only investigated in Western population, but also a research on the faecal bacterial dysbiosis in Chinese CD patients showing an increase of the richness *γ-Proteobacteria* (especially *Escherichia coli* and *Shigella flexneri*) and a reduced proportion of *Bacteroides* and *Firmicutes*[26]. Such differences were also observed by others applying terminal restriction fragment length polymorphism (T-RFLP) and fluorescent in situ hybridization (FISH) [27-29]. In murine models of IBD, *Bacteroidales* (*Bacteroides* sp., *Alistipes*, *Butyricimonas*, *Odoribacter*, and *Parabacteroides* sp.) and *Lactobacillus sp.* were predominantly associated with the DSS-induced colitic and healthy rats, respectively [30]. Obviously, there were significant differences between the experimental sets from which samples were sourced. This may be caused by many factors including genetics, variations in environmental conditions from different geographic locations, as well as the microbiological status of food and water. Despite these differences, most of the studies have shown an increase of some opportunistic pathogenic *Proteobacteria* and a decreased proportion of *Firmicutes* phylum in CD, UC, or IBD.

The role of the microbiota in the zebrafish larval TNBS model has not been previously described. Our results showed that the dominant bacterial species were altered in the larvae intestine with TNBS-induced IBD, which was characterized by an overrepresentation of *Proteobacteria* and a relative lack of *Firmicutes* phylum. We observed that *Limnobacter sp.*, *Comamonas sp.* and *Salmonella sp.*, major members of *Proteobacteria*, had a significantly elevated abundance and became to be dominant in the samples from TNBS-treated groups. This is consistent with previous reports in IBD, which suggests that the host-microbial interactions are evolutionarily conserved and bacterial communities within the zebrafish intestines contribute the same to IBD etiology as in mammals. This work thus highlights the potential use of zebrafish in the study of gut microbial contributions to the pathogenesis of IBD and also other intestinal disorders. In fact, the zebrafish has shown several unique advantages that make it superior to other animal model organisms for microbial investigation. To start with, the composition of the mucosal- and luminal-associated/faecal microbiota has been shown to be significantly different in human digestive tract [31,32]. Some believe the mucosal-associated microbiota seems of a closer link to the disease process than the faecal microbiota, as IBD is a disorder of mucosal inflammation. For a better understanding, characterisation of the mucosal-associated bacteria is therefore required. However, investigations are limited due to the difficulties of sampling of mucosal biopsy from healthy people. Besides, there is no conclusion whether the mucosal- or luminal-associated microbiota represents the accurate composition of the microbiota from patients with IBD. In contrast, our samples contain both the luminal- and mucosal-associated microbiota of the entire GI tract, which could reveal a better picture of the intestinal microbiotal composition. Furthermore, there was significant inter-individual variation in gut bacterial composition among both healthy and IBD groups in either humans or animal models research. The high inter-individual variability may cause confusion whether the microbiota shifts owing to the disease or the lifestyle and environmental changes. Whereas in zebrafish models, as each sample contains about 20 larvae, the individual differences could be greatly eliminated and more focusing on the differences in microbial communities between IBD groups and the healthy control. Finally, although studies have indicated a role for the microbiota in IBD development, to further understand this relationship between microbiota and host immunity and its degradation in inflammatory disease of the intestine, the next step must surely involve signaling pathways and molecular mechanisms through which the host recognizes gut microbiota and stimulates inflammatory processes. Rodent studies indicate that initial recognition of microbiota in the extracellular environment occurs via pathogen-recognition receptors (PRRs), which recognize microbial-associated molecular patterns (MAMPs) [33,34]. Some studies have shown that TLR4 knockout mice did not develop enterocolitis upon treatment with DSS and TLR4 antagonist antibody ameliorates inflammatory response in colitic mice [35,36]. In addition, a meta-analysis revealed that genetic variations in TLR4 presented a statistically significant risk of developing CD and UC [37]. It is reasonable to speculate that gut microbiota play a role in the development of IBD via TLR signaling pathways. In zebrafish models, reverse genetic analyses using target-selected mutagenesis or antisense morpholino oligonucleotides (MOs) provide additional means for identifying molecular mediators of host–bacterial relationships in the gut [38,39]. The completion of the zebrafish genome will facilitate these approaches and many more recently studies show the feasibility of studying host–microbial interactions in genetically engineered zebrafish.

## Conclusions

In summary, we represented for the first time the molecular characteristics of intestinal microbiota dysbiosis in larval zebrafish with TNBS-induced IBD-like colitis. The present study defined a reduced biodiversity of gut bacterial community in IBD-like colitis. The intestinal microbiota dysbiosis in zebrafish IBD-like models was characterized by an increase of *Proteobacteria* and a reduced proportion of *Firmicutes*. The major challenge here is elucidating whether alterations in the gut microbial composition represent cause, or consequence, of host inflammation and disease state in IBD. In deed, it could be hypothesize that the chemicals, eg, TNBS, oxazolone, or DSS, affect the microbiota composition and then alterations in the microbial community initiate mucosal immune-mediated inflammation via TLRs signaling pathways. It is possible that changes in gut microbial ecology are crucial determinants in the susceptibility to experimental enterocolitis. However, in the present study, we observed that the intestinal epithelial damage and the overproduction of inflammatory cytokine (TNF-α) appeared ahead of the intestinal microbiota shifts. This may suggest that the chemicals initiate inflammation and the progressive inflammatory damage to the host intestinal mucosa applies pressure on the intestinal microbiota that further shifts community structure. Or the host and the microbiota interact in both ways and there is a feedback loop that perpetuates the inflammation. In characterizing these changes in community structure and function, it may be possible to provide new clues into determining the aetiological mechanisms of IBD and alter these events to prevent or ameliorate the disease.

## Methods

### Ethics statement

All experiments with zebrafish were performed in strict accordance with the recommendations in the Guide for the Care and Use of Laboratory Animals of the National Institutes of Health. The protocols were approved by the Institutional Animal Care and Use Committee of Model Animal Research Center, Nanjing University (MARC-AP#: QZ01), in accordance with the Guideline on the Humane Treatment of Laboratory Animals in China and the Regulations for the Administration of Affairs Concerning Experimental Animals.

### Zebrafish maintenance and embryo collection

Wild-type (AB strain) zebrafish were reared at 28±0.5°C on a 14-h light/10-h dark cycle in a closed flow-through system in charcoal-filtered and fully aerated tap water according to standard procedures [40]. The fish were fed with commercial flakes twice daily.

Zebrafish embryos were collected from spawning adults in groups of about 16 males and 8 females in tanks overnight. Spawning was induced in the morning shortly after the light was turned on. Collected embryos were maintained in embryo medium (13.7 mM NaCl, 0.54 mM KCl, 1.3 mM CaCl_2_, 1.0 mM MgSO_4_, 0.25 mM Na_2_H PO_4_, 0.44 mM KH_2_ PO_4_, 0.42 mM NaHCO_3_) at 28.5°C. At 4–5 hours post-fertilization (hpf), those embryos that had developed normally and reached the blastula stage were selected under a dissecting microscope for subsequent experiments.

### Induction of IBD by TNBS exposure

A stock solution of 5% (w/v) 2, 4, 6-trinitrobenzenesulfonic acid (TNBS; Sigma, St Louis, USA) in embryo medium was used for the induction of IBD. Zebrafish from 3 days post fertilization (dpf) were randomly placed into groups of 15 larvae in 20 ml of exposure solution (embryo medium containing 0, 25, 50 and 75 μg/mL TNBS). The range of concentrations was selected based on previously ascertained range-finding studies and information from the available literatures [14,15]. A 90% (v/v) water change was performed each day starting at 3 pdf when larvae hatch from their chorions. Samples were collected at 4, 6 and 8 days postfertilization (dpf).

### Histology

Larval zebrafish from 4 dpf, 6 dpf and 8 dpf were anesthetized by immersion in 0.2 mg/ml 3-amino benzoic acid ethylester (MS222, Sigma). For histology, samples were fixed in Bouin’s Fixative overnight at 4°C and mounted in SeaPlaque 1% low-melting point agarose. Then samples were dehydrated through a standard series of alcohols and Histo-clear and embedded in paraffin. 5 μm sections were cut for staining with hematoxylin and eosin. Histological sections were imaged and photographed with an Olympus CX41 system microscope (Olympus USA, Center Valley, PA, USA) and the DS-5 M-L1 digital sight camera system (Nikon, Japan). The enterocolitis scores were quantified by an observer who was blinded to the prior treatment of the fish. And these data represent three independent experiments.

### Detection of goblet cells using AB-PAS staining

For goblet cell quantification, 5-μm paraffin sections were prepared as described in the Methods and stained sequentially with 1% Alcian blue pH 2.5 for 15 min, 1% aqueous periodic acid for 10 min and Schiff’s reagent for 10–15 min. Using this method, goblet cells stain blue. The number of goblet cells was counted manually along the length of the gut from the intestinal bulb to the anus.

### Immunofluorescence

Larvae at 4 dpf, 6 dpf and 8 dpf were fixed in 4% paraformaldehyde overnight at 4°C. Fixed larvae were soaked in 30% sucrose until they sink, transferred to embedding chamber filled with OCT Compound (Sakura Finetek USA, Inc, Torrance, CA, USA), snapped frozen in liquid nitrogen and stored at −80°C.

For immunofluorescence, 5-μm frozen sections were cut and blocked with 1% bovine serum albumin prior to being incubated with anti-TNF-α(IN), Z-Fish™, Catalog No. 55383P (1:150, 100 μg/400 μl, AnaSpec, Fremont, CA) overnight at 4°C. Sections were washed in PBS and incubated with Alexa Fluor 488-conjugated anti-mouse secondary antibodies (1:150, Invitrogen, La Jolla, CA) for 30 minutes at 4°C, followed by counterstained with DAPI (1:500). Sections were imaged and photographed with Leica TCS SP5 confocal scanning microscope (Leica Microsystems, Heidelberg GmbH, Mannheim, Germany). The intensity of TNF-α immunofluorescence was quantified for each treatment group, with a minimum of 6 samples per group, using color threshold and area measurements with AnalySis software.

### Microbial analysis by denaturing gradient gel electrophoresis (DGGE)

The DGGE analysis was carried out to identify the microbial community in the intestine and to study the potential changes between the different groups of zebrafish.

### Extraction of DNA and PCR amplification

Bacterial DNA was extracted from pools of 20 zebrafish larvae using the QIAamp DNA Stool Mini Kit (QIAGEN, Hilden, Germany) according to the manufacturer’s protocol, and stored at −20°C until use.

PCR was performed on an Applied Biosysterm 2720 Thermal Cycler as a touchdown PCR. The hypervariable V3 region of the 16S ribosomal DNA gene was amplified using polymerase chain reaction (PCR) with forward primer (GC357f 5′CGCCCGGGGCGCGCCCCGGGCGGGGCGGGGGCACGGGGGGATTACCGCGGCTGCTGG3′) and reverse primer (518r 5′CCTACGGGAGGCAGCAG3′). The PCR reaction mixtures consisted of 2 μl of extracted bacterial DNA, 5 μl of 10×PCR buffer, 1 μl of dNTP mixture (2.5 mM each), 1 μl of each primer (10 pM), 0.5 μl of *Taq*-Polymerase (5 U/μl) and sterile water to final volume of 50 μl. The cycling program was as follows: predenaturation at 94°C for 5 min, followed by 20 cycles of 94°C for 30 s, 65°C for 30 s decreased by 0.5°C for each cycle, and 68°C for 30 s, after which 10 additional cycles of 94°C for 30 s, 55°C for 30 s, and 68°C for 30 s were carried out, and a final extension at 68°C for 7 min, soak at 4°C.

Integrity of PCR products was determined by running agarose gel electrophoresis, and the quantity was determined using QubitTM fluorometer (Invitrogen, NY, USA).

### Denaturing gradient gel electrophoresis

DGGE was performed on the PCR products from DNA samples using 16 cm × 16 cm ×1 mm gels with a DCode Universal Mutation Detection System (Bio-Rad, Hercules, CA). A 35-50% urea and formamide denaturing gradient and 8% polyacrylamide gel (37.5:1 acrylamide-bisacrylamide) were used. The gradient was prepared using the gradient delivery system (Bio-Rad), following the manufacturer’s protocol. A 100% denaturant solution contained 7 M urea and 40% formamide. Gels were run in 1×TAE (20 mM Tris, 10 mM acetate, 0.5 M EDTA, pH 7.4) at 60°C, first at 200 V for 10 minutes and then at 120 V for 7.5 hours. The resulting gels were stained with SYBR Green I (Invitrogen) for 30 min, visualized and photographed using the Gel Doc EQ system (Bio-Rad, USA). All gels were normalized using a reference sample with bands distributed throughout the whole gel.

### Analysis of DGGE profile

Gel images were aligned using Adobe Photoshop CS5 by running common samples on both outer sides of each gel, to allow comparison of two gels in one profile. DGGE profiles were analysed using Quantity One software (version 4.6; Bio-Rad Laboratories, Hercules, CA). The lanes were identified, and their background intensities were removed using the rolling disk method described in the program. Then bands were detected automatically by the software, followed by manual correction if necessary, and they were matched at 0.5% tolerance level. The tolerance level is the minimum spacing that the matching model expects to find between unique bands, and it is expressed as a percentage of lane height. The relative quantity of bands is expressed as a proportion (%) relative to the sum of the intensities of all of the bands in the same lane. A similarity matrix was computed by comparing the profiles of lanes, and the percentage similarity was expressed as the Dice coefficient. The presence or absence of a band in a lane was considered. Identical profiles have a percentage similarity of 100. Unweighted pair group method using arithmetic averages (UPGMA) was used to compare the similarity of samples in a dendrogram. The general diversity of bacterial communities was calculated by generating Shannon’s index of diversity on quantitative information [41].

### Sequencing of DGGE bands

Bands of interest from DGGE gels were excised and immersed in 20 μl of sterile water and left overnight at 4°C. 2 μl of eluted DNA from each band was used as template for PCR re-amplification with the forward primer (without GC clamp) (357f 5′- ATTACCGCGGCTGCTGG -3′) and the reverse primer (518r 5′-CCTACGGGAGGCAGCAG-3′). PCR was performed in a 50 μl reaction mixture including 2 μl of template DNA, 5 μl of 10×PCR buffer, 1 μl of dNTP mixture (2.5 mM each), 1 μl of each primer (10 pM), 0.5 μl of Taq-Polymerase (5 U/μl) and 39.5 μl sterile water. Amplification was performed under the following conditions: 94°C for 5 min, 20 cycles of 94°C for 30s, 65°C for 30s decreased by 0.5°C for each cycle, and 68°C for 30 s, additional 15 cycles of 94°C for 30 s, 55°C for 30 s, and 68°C for 30 s, with a final extension at 68°C for 7 min.

After the PCR products were purified (QIAquick PCR Purification Kit, QIAGEN) and quantified (Qubit fluorometer, Invitrogen), the sequence analysis of the products was carried out using the Sanger’s method on an ABI 3730 automated sequencing system. The sequences obtained were then aligned with NCBI GenBank databases using the BLAST tool. The phylogenetic tree was constructed using the MEGA 4.0 program in the method of neighbor-joining based on evolutionary distances.

### Quantitative real-time PCR analysis

Bacterial species that characterize the predominant intestinal dysbiosis in zebrafish larvae with TNBS-induced enterocolities derived from the DGGE comparative analyses were quantified by quantitative PCR using the 7300 Real-Time PCR System (Applied Biosystems, USA). A reaction mixture (20 μl) consisted of 1 μl of DNA (10 ng), 0.4 μl of each primer, 10 μl 2×SYBR. The primers and probes based on 16S rRNA gene sequences were chosen to target total bacteria, *Lactobacillus group*, the dominant group of Firmicutes, *Enterobacteriaceae family* and *Burkholderia species*, the main Proteobacteria phylum in zebrafish gut. Total bacterial 16S rRNA gene copies were quantified with primers (Bact1369; 5′CGGTGAATACGTTCYCGG3′and Prok1492; 5′GGWTACCTTGTTACGACTT3′). PCR was performed with an initial denaturation step of 95°C for 3 min, followed by 40 cycles of 95°C for 15 s, 56°C for 30 s and 72°C for 30 s. *Lactobacillus group* were quantified using the combination of forward, (LAC1; 5′AGCAGTAGGGAATCTTCCA3′), and reverse primer, (Lab0677; 5′CACCGCTACACATGGAG3′) in a cycling program where after the initial denaturation 95°C for 3 min, 40 cycles were applied at 95°C for 30 s, and binding and extension at 60°C for 1 min. Primer (Eco1457F; 5′CATTGACGTTACCCGCAGAAGAAGC3′) combined with primer (Eco1652R; 5′CTCTACGAGACTCAAGCTTGC3′) were used for the quantification of *Enterobacteriaceae family* with the following conditions: an initial DNA denaturation step at 95°C for 5 min, followed by 40 cycles of denaturation at 95°C for 15 s, and primer annealing and extension at 72°C for 30 s. *Burkholderia species* were quantified using the forward primer (Burk3; 5′CTGCGAAAGCCGGAT3′) and the reverse primer (BurkR; 5′TGCCATACTCTAGCYYGC3′) with the following cycling conditions: predenaturation at 95°C for 4 min; 60 cycles of 94°C for 1 min, 62°C for 90 s decreased by 1°C for every fifth cycle, after which 25 additional cycles were carried out at 58°C, and 72°C for 2 min, and a final extension at 72°C for 10 min. Data analysis was proceeded with Sequence Detection Software version 1.6.3 ( Applied Biosystems). All reactions were performed in triplicate. Specific bacteria 16S rRNA gene amount was normalized to total bacteria 16S rRNA. Quantification values were represented as mean (SEM) log 16S rRNA gene copies per 10 ng of bacterial genomic DNA.

### Statistical analysis

Biochemical measurements were performed at least in duplicate. Quantitative histological analyses were performed by a blinded scorer. Results are presented as mean ± standard error of the mean. Survival curve comparison calculations used the Gehan-Breslow-Wilcoxon test. Two-way anova was applied to analyze the data to understand the combined effect of the two factors - time and treatment. Bonferroni multiple comparison post hoc tests were used to find the significant differences between the means at a particular time point⁄treatment. Pearson correlation, α =0.05, was used to assess linear relationships between enterocolitis score/inflammatory cytokine expression level and intensity/diversity in gut microbiota. All statistical analyses were performed with Graph-Pad Prism version 5.0 (GraphPad Software, San Diego, CA), and the significant differences are reported at *P* < 0.05.

### Nucleotide sequences accession number

The sequences of 16S rRNA gene obtained in this study have been deposited in the GenBank database (EMBL, U.K.) under accession numbers KF515539-KF515557.

## Competing interests

The authors declared that they have no competing interests.

## Authors’ contributions

QH carried out the zebrafish model-building, the sequence analysis and drafted the manuscript. LW participated in the Immunofluorescence analysis. FW and CYW participated in the sequence alignment. CT participated in the histological analysis. QRL and JSL conceived of the study, and participated in its design and coordination and helped to draft the manuscript. All authors read and approved the final manuscript.
